# Complete Genome Sequence of *Acinetobacter baumannii* ZW85-1

**DOI:** 10.1128/genomeA.01083-13

**Published:** 2014-01-23

**Authors:** Xin Wang, Zhewen Zhang, Qiong Hao, Jiayan Wu, Jingfa Xiao, Huaiqi Jing

**Affiliations:** a National Institute for Communicable Disease Control and Prevention, Chinese Center for Disease Control and Prevention, State Key Laboratory for Infectious Disease Prevention and Control, Beijing, China; b CAS Key Laboratory of Genome Sciences and Information, Beijing Institute of Genomics, Chinese Academy of Sciences, Beijing, China; c Ningxia Hui Autonomous Region Centre for Disease Control and Prevention, Yinchuan, China

## Abstract

*Acinetobacter baumannii* is an aerobic, nonmotile Gram-negative bacterium that causes nosocomial infections worldwide. Here, we report the complete genome sequence of *Acinetobacter baumannii* strain ZW85-1 and its two plasmids. One of the plasmids carries genes for NDM-1, which can hydrolyze a wide range of antibiotics.

## GENOME ANNOUNCEMENT

*Acinetobacter baumannii* is an important nosocomial pathogen which is distributed in a variety of environments in the hospital (1). It is difficult to treat infections caused by *Acinetobacter baumannii* because of its multiple-drug resistance (2–4). To gain insight into the genome structure and resistance gene environment of *Acinetobacter baumannii*, we determined the draft genome sequence of *Acinetobacter baumannii* strain ZW85-1, which was isolated from diarrheal patient feces and shows the presence of the New Delhi metallo-β-lactamase (NDM-1).

The genome of ZW85-1 was sequenced by use of 454 GS FLX Titanium pyrosequencing (Roche) with 51-fold coverage. Sequencing yielded 622,283 reads, and these reads were assembled into 56 large contigs (length >500 bp) using the Newbler 2.3 assembly software program (Roche). Gaps between large contigs were filled by sequencing PCR products by means of an ABI 3730 capillary sequencer. The sequences from ABI 3730XL sequencing and large contigs were assembled using Phred/Phrap/Consed (5) software. The open reading frames (ORFs) were predicted by Glimmer 3.0 (6) and GeneMarkS (7). tRNA genes were located using tRNAscan-SE (8). RNAmmer1.2 (9) was used to find 5S, 16S, and 23S rRNA in full-genome sequences. Functional classification was performed by aligning predicted proteins to the Clusters of Orthologous Groups (COG) database (10). All predicted genes were compared to a nonredundant (nr) protein database in NCBI using BLASTX (11), with *E* values of ≤1e−5 and identity of ≥30%. Metabolic pathways were analyzed by a single-directional best-hit method on the KEGG web server (http://www.genome.jp/kegg/). The comparative genomic analysis of the ZW85-1 genome with 15 other *Acinetobacter baumannii* strains with complete genome sequences in GenBank was performed with PGAP (12).

*Acinetobacter baumannii* ZW85-1 has one chromosome, which is 3,763,012 bp in size with a GC content of 39%, and two plasmids, ZW85p1 and ZW85p2, consisting of 48,368 bp (GC content 40.7%) and 113,866 bp (GC content 41.9%), respectively. The chromosome has 3,465 predicted coding sequences (CDS), 6 copies of 16S-23S-5S rRNA operons, and 69 tRNA genes, while ZW85p1 and ZW85p2 contain 52 and 119 protein-coding genes, respectively. As to the chromosome genes, over 72% were assigned to specific COG, and approximately 52% of the genes were assigned to a KEGG orthologous number and involved in 163 predicted metabolic pathways. By comparing the ZW85-1 genome to those of 15 other *Acinetobacter baumannii* strains, we found 134 unique genes in ZW85-1. The majority of these genes encoded hypothetical proteins, and one encoded a small multidrug resistance protein which is a membrane transporter of cations and cationic drugs.

A BLAST search showed that approximately 47 kb of the ZW85p1 plasmid displayed 100% identity with the plasmid sequence of *Acinetobacter lwoffii* pNDM-BJ01 (13). ZW85p1 contains both a bla_NDM-1_ gene and a type IV secretion system gene cluster, as reported for *Acinetobacter lwoffii* pNDM-BJ01. Many *bla*_NDM-1_-carrying *Acinetobacter baumannii* strains have been reported (14–17), but the ZW85-1 strain, which is a clinical isolate of the *Acinetobacter baumannii* strain carrying the NDM-1 gene in China, is the first to be completely sequenced. This information elucidates the gene environment of *bla*_NDM-1_ and will enhance the further analysis of the putative origin of the ZW85p1 plasmid.

### Nucleotide sequence accession numbers.

The genome sequences of *Acinetobacter baumannii* ZW85-1 and its two plasmids have been deposited in NCBI GenBank under the accession numbers CP006768, JN377410, and CP006769.
